# Intra-assessor reliability and measurement error of ultrasound measures for foot muscle morphology in older adults using a tablet-based ultrasound machine

**DOI:** 10.1186/s13047-022-00510-1

**Published:** 2022-01-25

**Authors:** Lydia Willemse, Eveline J. M. Wouters, Martijn F. Pisters, Benedicte Vanwanseele

**Affiliations:** 1 Department of Health Innovations and Technology, Fontys University of Applied Sciences, PO Box 347, 5600 AH Eindhoven, Netherlands; 2 Department of Movement Sciences, KU Leuven, Tervuursevest 101 - box 1500, 3001 Leuven, Belgium; 3 Tranzo, School of Social and Behavioral Sciences, Tilburg University, PO Box 90153, 5000 LE Tilburg, Netherlands; 4 Department of Rehabilitation, Physiotherapy Science & Sport, UMC Utrecht Brain Center, Utrecht University, PO Box 85500, 3508 GA Utrecht, The Netherlands; 5 Center for Physical Therapy Research and Innovation in Primary Care, Julius Health Care Centers, PO Box 85500, 3508 GA Utrecht, Netherlands

**Keywords:** Intrinsic foot musculature, Ultrasound, Older adults, Reliability, Measurement error

## Abstract

**Background:**

To gain insight into the role of plantar intrinsic foot muscles in fall-related gait parameters in older adults, it is fundamental to assess foot muscles separately. Ultrasonography is considered a promising instrument to quantify the strength capacity of individual muscles by assessing their morphology. The main goal of this study was to investigate the intra-assessor reliability and measurement error for ultrasound measures for the morphology of selected foot muscles and the plantar fascia in older adults using a tablet-based device. The secondary aim was to compare the measurement error between older and younger adults and between two different ultrasound machines.

**Methods:**

Ultrasound images of selected foot muscles and the plantar fascia were collected in younger and older adults by a single operator, intensively trained in scanning the foot muscles, on two occasions, 1–8 days apart, using a tablet-based and a mainframe system. The intra-assessor reliability and standard error of measurement for the cross-sectional area and/or thickness were assessed by analysis of variance. The error variance was statistically compared across age groups and machines.

**Results:**

Eighteen physically active older adults (mean age 73.8 (SD: 4.9) years) and ten younger adults (mean age 21.9 (SD: 1.8) years) participated in the study. In older adults, the standard error of measurement ranged from 2.8 to 11.9%. The ICC ranged from 0.57 to 0.97, but was excellent in most cases. The error variance for six morphology measures was statistically smaller in younger adults, but was small in older adults as well. When different error variances were observed across machines, overall, the tablet-based device showed superior repeatability.

**Conclusions:**

This intra-assessor reliability study showed that a tablet-based ultrasound machine can be reliably used to assess the morphology of selected foot muscles in older adults, with the exception of plantar fascia thickness. Although the measurement errors were sometimes smaller in younger adults, they seem adequate in older adults to detect group mean hypertrophy as a response to training. A tablet-based ultrasound device seems to be a reliable alternative to a mainframe system. This advocates its use when foot muscle morphology in older adults is of interest.

**Supplementary Information:**

The online version contains supplementary material available at 10.1186/s13047-022-00510-1.

## Background

Less propulsive gait and diminished balance capabilities, being consequences of the normal ageing process [1, 2], are associated with an increased likelihood of falling in older adults [1, 3–6]. Plantar intrinsic foot muscles (PIFMs) play an important role in these two features of gait, at least when they are unaffected [7–9]. For instance, propulsion is aided by the PIFMs by stiffening the foot during the push-off phase of walking, hence contributing to the effective force transmission onto the ground [7]. The PIFMs also act to stabilize the foot arch, which is imperative for sound postural balance [8, 9].

Concurrent with the PIFMs’ related mobility decline, a decreased capacity of the PIFMs to produce force has been observed in older adults [10]. In this population, toe flexor weakness is associated with a higher probability of falling [11]. Consequently, toe flexor strengthening is often one of the goals in fall prevention interventions [12]. However, toe flexor strength is the combined result of contraction of intrinsic foot muscles (i.e., origin and insertion in the foot) and extrinsic foot muscles (i.e., origin proximal to ankle joint, insertion in the foot), both having a shared as well as a distinct function [9, 13]. It is thus important to distinguish the PIFMs as a separate group of foot muscles, as well as to distinguish individual PIFMs, in order to gain more insight in the unique role of PIFMs in fall-related mobility parameters. These insights may lead to the enhancement of related treatment.

Recently, some studies investigated the role of individual PIFMs in foot function [14] or evaluated a PIFM strengthening intervention [15], using a measure for toe flexor strength or strength capacity. However, directly assessing the strength of individual foot muscles is unviable in vivo because of the redundant combinations of intrinsic and extrinsic foot muscles’ contractions resulting in the same net force [16]. Therefore, measuring flexor strength of plantar foot muscles in units of force is restricted to measuring net toe flexor or toe grip force produced by the PIFMs in conjunction with the extrinsic foot muscles [16]. To overcome this limitation, ultrasound has been applied to study individual foot muscles (i.e., both intrinsic and extrinsic) [17–19]. This imaging technique is used to obtain the dimensions of these muscles, as an estimate of its capacity to exert force. The validity of this approach is confirmed by the observation that both the cross-sectional area (CSA) and the thickness of the PIFMs correlate well with maximum toe flexor force [10, 20–22].

Although magnetic resonance imaging (MRI) is considered the gold standard in the assessment of muscle morphology, ultrasonography is often preferable in both clinical and research settings [23]. In comparison with an MRI machine, an ultrasound machine is more accessible, portable and has superior temporal and spatial resolution when used to image superficial structures [23, 24]. The ongoing advancement of pocket-sized ultrasound equipment advocates the utility even more [25]. Despite the eminent tissue differentiating capabilities of MRI [26], ultrasound derived measures for lower extremity muscle morphology correlate well with values obtained by using MRI [26–29]. In contrast to MRI, ultrasonography enables the operator to capture a muscle’s contraction in a cine-loop, which facilitates the post-processing identification of a muscle’s circumference [30].

Determining the morphology of specifically the PIFMs, as part of the foot muscles, using ultrasound images is, however, challenging. This is due to the complex anatomical architecture of each of these muscles [31] and their non-parallel arrangement over several muscle layers [32]. Nevertheless, in general, studies revealed excellent inter- and intra-operator reliability and acceptable measurement errors for the ultrasound assessment of the thickness and CSA of PIFMs in younger adults [21, 33–36]. In addition, a study [37] that compared the reliability across machines for one of the PIFMs (i.e., abductor hallucis), demonstrated at least good reliability, even when using a laptop-based machine. These findings indicate the potential of ultrasonography to discriminate between individuals and to measure changes over time [38].

However, these measurement properties (i.e., reliability and measurement error) cannot be simply generalized to older adults for two reasons. Most importantly, physiological changes that occur with ageing, such as a higher degree of intramuscular adiposity and connective tissue [39] or increased presence of callus [40], may interfere with image quality and thus may limit the accuracy of muscle morphology measurements [41, 42]. Furthermore, the reliability, as expressed in the intra-class correlation coefficient (ICC), is mathematically dependent on the biological variability between subjects [38] and this may differ between younger and older adults. Although ultrasonography has been shown to be a reliable instrument to measure quadriceps and gastrocnemius morphology in older adults [43], this is still unknown for the foot muscles. These muscles, including the PIFMs, extrinsic toe flexor muscles, but also extrinsic in- and evertors, should be jointly assessed together with the plantar fascia (PF), considering the synergistic contribution of this group of foot tissues to foot function [17–19]. The reliability of ultrasonography to assess the morphology for these foot muscles and the PF needs to be determined in order to judge the potential of this instrument to be used in future research concerning, for instance, the role of the foot muscles in relation to fall risk-related mobility parameters. For this future purpose, a single operator performing the ultrasound scans is recommended, as this is expected to result in more reliable measures [43].

Therefore, the main goal of this study was to investigate the intra-assessor reliability and measurement error of ultrasound measures for the morphology of selected foot muscles and the PF in older adults using a tablet-based ultrasound machine. In addition, we assessed a younger population using the same ultrasound machine and a mainframe machine to explore factors that could underlie these measurement properties. To investigate the effect of age, we compared the measurement error between older and younger adults using the tablet-based machine. To investigate if improved repeatability in older adults can be expected when changing to a mainframe machine, we compared the measurement error between the tablet-based and the mainframe machine in younger adults.

## Methods

### Study design

The design used was a blinded single assessor test–retest reliability study.

### Ethical considerations

The medical ethical committee of Maxima MC declared that ethical approval was not required for this study protocol (N19.105). Written informed consent was obtained before the start of data collection.

### Study population

#### Recruitment

A sample of 18 older adults was recruited in the region of Eindhoven, The Netherlands, via advertisement in senior housing complexes and by sending a recruitment leaflet per e-mail to the social network of the investigator. Ten younger adults were recruited through personal communication within the University of Applied Sciences, Eindhoven, The Netherlands. Due to a lack of consensus on the required number of participants to achieve reliable measurement properties [38], the sample sizes were based on comparable studies [33, 34].

#### Selection procedure

Individuals were eligible for participation in the older adult group if they were at least 65 years of age, in accordance with the categorization of the World Health Organization [44]. To participate in the younger adult group, volunteers had to be between the ages of 18 and 45 years, as lower extremity muscles start to atrophy after the age of approximately 45 years [45]. Further, volunteers had to be free of any known condition or disease affecting foot muscles and had to be able to ambulate ten meter without using a walking aid in order to represent a mobile population. Volunteers were excluded from study participation if they reported bilateral musculoskeletal injuries or bilateral symptoms distal to the knee (i.e., current musculoskeletal pain or overuse symptoms, orthopedic surgery or acute injury within the past 5 years, amputation) or if mobility or lower extremity motor function was likely to be affected by medical conditions (i.e., neurological condition, systemic disease, cardiovascular or pulmonary disease).

### Measurement procedure

**Fig. 1:**
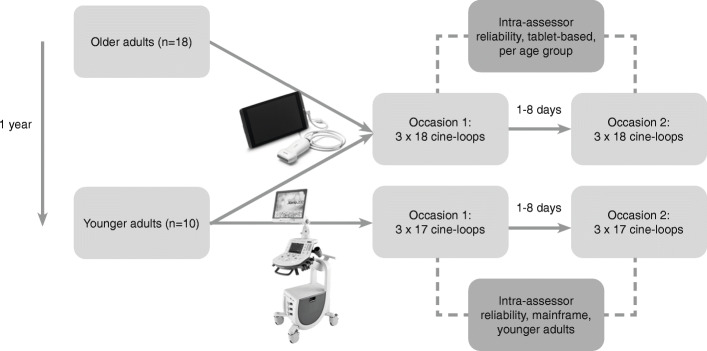
Schematic overview of the study set-up. The dotted lines link the measurement occasions that were selected as the repeated measurements for the reliability analysis

The measurement set-up is schematically depicted in Fig. 1. In a first period, images of foot muscles and PF were acquired in the older participants using a tablet-based ultrasound machine only. Thereafter, we decided to repeat the protocol in younger adults to explore factors that could underlie the established measurement properties. Approximately one year after the data collection period in older adults, the data were collected in the younger participants using both the tablet-based and a mainframe ultrasound machine for all measurements. This allowed us to investigate the influence of age on the measurement error and also to see if improved image quality, as expected from the mainframe machine, would reduce the measurement error. The ultrasound images in each participant were collected on two separate measurement occasions, at least one day apart [33], with a maximum of 8 days apart, assuming that foot muscle and PF morphology remains stable within this period. Participants were instructed not to engage in vigorous physical activities in the three days prior to the measurement sessions to avoid exercise-induced swelling of the muscles. The time of day was kept constant over the repeated measurements within participants. The older adults were measured at home. The younger adults were invited to the movement analysis laboratory at Fontys University of Applied Sciences (Eindhoven, the Netherlands).

At the first measurement occasion, demographics and characteristics were collected that are related to foot muscle and PF morphology or its ultrasound measurement reliability. Body length and weight were assessed manually in the older adults (213i and 750, Seca co., Hamburg, Germany) and electronically in the younger adults (DS-103, Dong Sahn Jenix co., Seoul, Korea). The participants were also asked about their physical activity behavior.

The ultrasound scans were performed by the investigator (LW), having a master degree in human movement sciences and several years of experience in teaching foot and ankle anatomy to podiatry students. She underwent a 10-month training program in imaging the foot muscles and PF prior to the study, without having previous experience in ultrasound imaging. The training started with three technical lectures and an individual training session from a formal ultrasound teacher specialized in musculoskeletal ultrasound. Throughout the training period, a few instructional sessions were supervised by either a physiotherapist experienced in clinical musculoskeletal ultrasonography or by a researcher experienced in imaging the foot muscles. The remainder of the training consisted of unsupervised sessions in which the proposed scan protocol [33, 34] was practiced on younger adult volunteers alternated by interpreting the ultrasound images using an interactive anatomy atlas of MRI images, cadaveric videos and schematic illustrations. After the training, a pilot study in younger adults revealed intra-assessor ICC and limits of agreement (LoA) values that were overall comparable to the ones found in a previous study with an experienced operator [33]. This was considered sufficient to start the data collection. Additionally, the collected data in the current study were examined for any inevitable ongoing improvement in the operator’s skills, which is explained in more detail in the statistical analysis paragraph below.

In the older and younger participants, the foot tissues were imaged using a tablet-based ultrasound system (Lumify, Philips Ultrasound, Inc., Bothell, USA) consisting of a 4–12 MHz broadband linear array transducer with a footprint length of 34 mm, the Lumify app and a Samsung Galaxy S4 tablet (Samsung Electronics co., Suwon, South Korea). Because of practical feasibility reasons, the scan protocol was repeated only in the younger participants using a mainframe system (Xario 200 g, Canon, Tochigi, Japan) with a 5–14 MHz linear array transducer (PLU-1005BT, Toshiba, Tochigi, Japan) with a footprint length of 58 mm. The order of the systems used was randomly chosen for each measurement occasion.

**Fig. 2:**
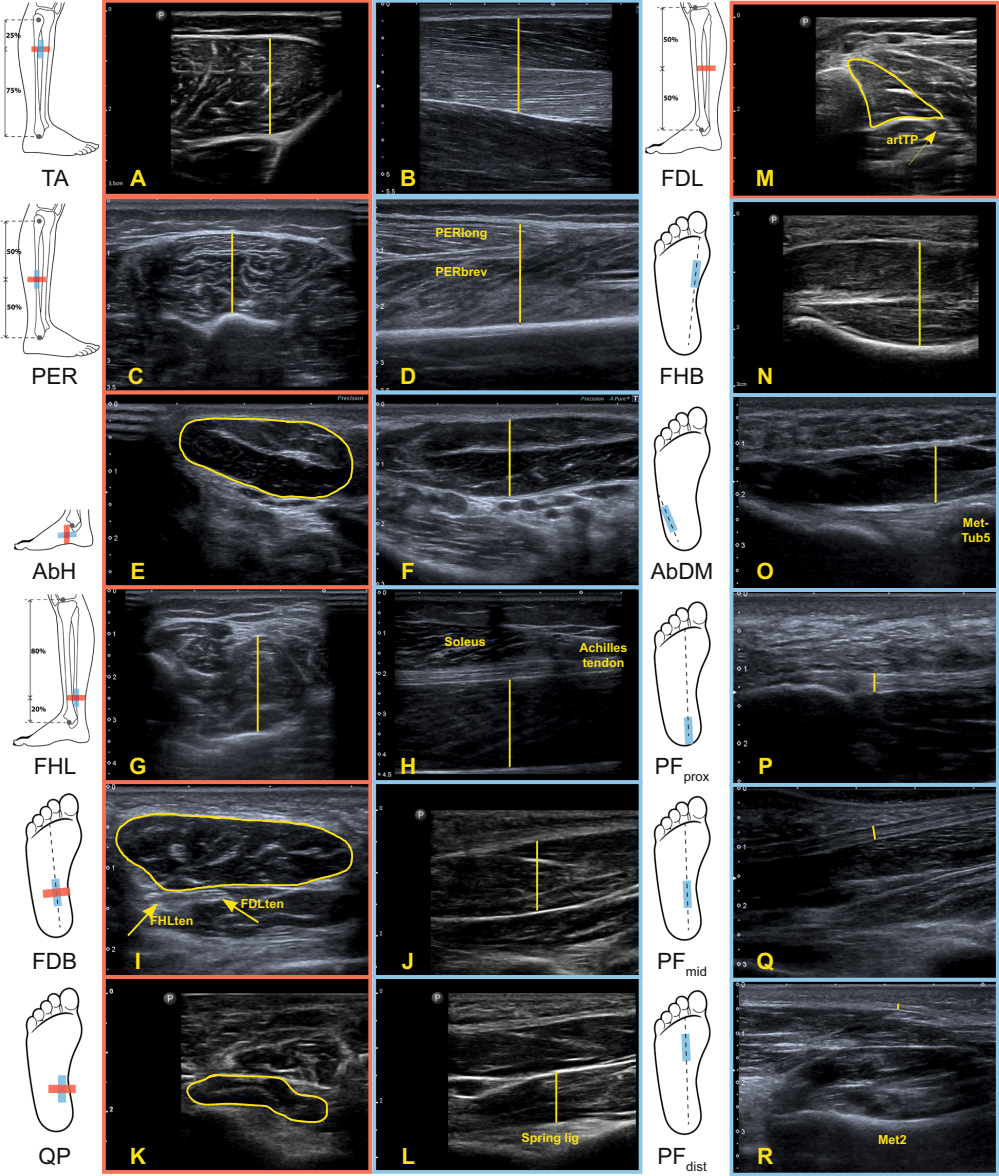
Probe position and representative ultrasound appearance for transverse (red) and longitudinal (blue) images of the selected foot muscles and plantar fascia. Ultrasound images shown are obtained with both the tablet-based (**A, J-N**) and the mainframe machine (**B-I, O-R**). TA: m. tibialis anterior, PER: m. peroneus brevis (brev) + longus (long), AbH: m. abductor hallucis, FHL: m. flexor hallucis longus, FDB: m. flexor digitorum brevis, QP: m. quadratus plantae, FDL: m. flexor digitorum longus, FHB: m. flexor hallucis brevis, AbDM: m. abductor digiti minimi, PF: plantar fascia, ten: tendon, lig: ligament, art: artery, MetTub5: 5th metatarsal tuberosity, Met2: 2nd metatarsal bone

The dominant stance limb, decided by asking the participant to stand on one leg, or the asymptomatic limb, in case of unilateral symptoms, was scanned. Foot structures that were imaged consisted of intrinsic foot muscles (i.e., abductor hallucis (AbH), flexor digitorum brevis (FDB), quadratus plantae (QP), flexor hallucis brevis (FHB), abductor digiti minimi (AbDM)) and extrinsic foot muscles (i.e., tibialis anterior (TA), peroneus longus together with the peroneus brevis (PER), flexor digitorum longus (FDL), flexor hallucis longus (FHL)) and PF. TA, PER, FDL and AbH were imaged while the participants were in a supine position, their knee slightly bent and their distal thigh resting on a cushion, preventing compression of the lower leg muscles. To image FHL, FDB, QP, FHB, AbDM and PF, the participants lay in a prone position, their foot hanging freely off the plinth and their distal shank resting on a cushion. Using anatomical landmarks, washable lines were drawn on the skin to guide the placement of the transducer. The scan protocol was adopted from previous studies [33, 34]. Based on our own pilot testing, we decided to image FHL slightly proximal to the ankle joint and TA at 25% of lower leg length. Figure 2 illustrates the transducer position and Additional file 1 provides a detailed scan protocol. Considering their shape, we decided to image all muscles, except for the FDL, and the PF in the longitudinal plane and selected muscles (TA, PER, AbH, FHL, FDB, QP, FDL) additionally in the transverse plane. Imaging QP in the transverse plane was omitted in the protocol for younger adults, because of the indefinite appearance of QP in the transverse images. This was most probably due to the non-parallel orientation of QP and its surroundings and was not expected to be an effect of age.

A generous amount of water-based coupling gel was applied between transducer and skin to obtain a clear image while avoiding compression of the tissue of interest. The probe was held perpendicular to the tissue border to achieve optimal appearance of the tissues of interest. The depth, focal point and gain were adjusted for each participant and each tissue to optimize image quality. In case of difficulties with identifying individual muscles and whenever possible, participants were asked to perform a specific movement to provoke a contraction that assisted the offline identification of the muscle’s border at the time of processing [46]. The contraction as well as the relaxed state of the muscle was captured in the same cine-loop of up to 5 s duration. Once the cine-loops were acquired for each muscle and the PF, the protocol was repeated twice resulting in three trials for each morphology measure. To minimize the discomfort for the participant of lying still, an efficient workflow was accomplished by fixing the order of tissues to be imaged. However, to avoid systematic interference (e.g., due to fatigue or familiarization) with the ultrasound measures, the starting position of the participant (i.e. supine or prone) was randomly chosen for each measurement occasion.

At the end of the session, the drawn scanning lines were removed from the skin. The captured videos were stored offline for the post-processing measurements. The whole procedure was repeated at the second measurement occasion, 1–8 days later.

### Data processing

The assessor who performed the post-processing to determine the morphology of the foot muscles and the PF was the same person who acquired the images (LW). Cine-loops were post-processed per trial per participant several days to several weeks after the images were collected. To avoid recall bias, no more than one trial per participant was processed per day, followed by at least two trials of other participants. This ensured that no less than 34 other values were assigned before another trial of the same participant was administered. Muscle dimensions obtained in previous trials were not presented to the assessor to further minimize the risk of recall bias.

Image J software (National Institute for Health, Bethesda, MD, USA) was used for the offline processing of data. Within the cine loop, the best quality frame was selected in which the muscle was in a relaxed state. To measure the thickness of the muscles and the PF, the built-in digital caliper was applied on all longitudinal images and the transverse images of TA, PER and FHL. The thickness of a muscle was represented by the vertical distance between the muscle’s epimysium, while the thickness of the PF was determined by the perpendicular distance between the deeper and superficial fascia borders. The CSA of FDL, AbH, FDB and QP was measured by delineating all intramuscular tissue using the polygon or freehand tool. Care was taken not to include hyperechoic surroundings of the muscles (e.g., epimysium or fascia) in the measurements for muscle morphology. The mean of three measurements (i.e., trials) for each morphology measure per occasion was administered as the final measure for further analysis.

### Statistical analysis

SPSS 25.0 (IBM, Chicago, IL, USA) was used for the statistical analysis. The study populations’ characteristics were specified by describing gender, age, body length, body weight, BMI, daily time spent on their feet and whether or not the global recommendations on physical activity for health were met [44]. Measurement characteristics included whether the dominant stance leg was measured, actual number of days between the measurement occasions and the difference in time of day between the measurement occasions.

ICC (3,1; absolute agreement) for a single measurement was estimated by intra-assessor reliability analysis of the repeated ultrasound measurements (occasions) based on a 2-way mixed-effects model. Cut-off values were used to interpret the ICC as a measure for reliability (i.e., < 0.50: ‘poor’, 0.50–0.75: ‘moderate’, 0.75–0.90: ‘good’ and > 0.90: ‘excellent’) [47]. The standard error of measurement (SEM) and smallest detectable change (SDC) were calculated using the error variance, estimated using a 2-way mixed-effects model via the restricted maximum likelihood approach, according to the formulae:

1 $$ {SEM}_{agreement}=\sqrt{{\sigma_{so.e}}^2} $$

2 $$ {SDC}_{agreement}=1.96\times \sqrt{2}\times {SEM}_{agreement} $$

where *σ*_*so*. *e*_^2^ is the error variance consisting of variance due to both systematic and random error.

All measurement properties (i.e., ICC, SEM, and SDC) were calculated separately for 1) older and 2) younger adults using the tablet-based machine and 3) for younger adults using the mainframe machine (Fig. 1). ‘Occasion’ was the only component that was varied across the repeated measurements.

In order to explore a possible learning curve, the older adults were divided over three sets of participants based on the chronological order of the first ultrasound measurement occasion [48]. The SEM for each morphology measure was calculated for each set of participants. When the SEM dropped with more than 50% from the one set to the next set of participants, without showing a previous increase, it was decided that the operator was still learning. The measurement properties (i.e., ICC, SEM, SDC), for the morphology measure(s) where this applied to, were then determined with the exclusion of the respective set(s) of participants.

Statistically (α = 0.05), 1) the SEMs for the tablet-based ultrasound measurements were compared between the older and younger adults and 2) the SEMs in the younger adults were compared between the tablet-based and the mainframe machine by applying the ‘variance ratio approach’ and the ‘paired approach’, respectively [49].

## Results

Table 1 shows the characteristics for the study populations and the measurements. Eighteen older adults with a mean age of 73.8 (SD: 4.9) years, and ten younger adults with a mean age of 21.9 (SD: 1.8) years participated in the study. The older adults had a higher body weight and BMI compared to the younger adults (mean body weight: 75.9 (SD: 13.5) kg vs. 63.8 (SD: 10.9) kg, *p* < 0.05; mean BMI: 26.3 (SD: 3.2) kg/m^2^ vs. 21.4 (SD: 2.5) kg/m^2^, *p <* 0.05). Other participant characteristics were not statistically different between the age groups, nor were the measurement characteristics.

**Table 1:** Participant and measurement characteristics for the older and younger group of participants

	Older (*n* = 18)	Younger (*n* = 10)
Gender		
Male	8 (44%)	4 (40%)
Female	10 (56%)	6 (60%)
Age in years ^a^	73.8 (4.9)*	21.9 (1.8)
Body length in cm ^a^	169 (11)	172 (7)
Body weight in kg ^a^	75.9 (13.5)*	63.8 (10.9)
BMI in kg/m^2 a^	26.3 (3.2)*	21.4 (2.5)
Daily time spent on feet		
< 1 h	–	–
1–4 h	9 (50%)	2 (20%)
4–8 h	6 (33%)	7 (70%)
≥ 8 h	3 (17%)	1 (10%)
Physical activity behavior as recommended	16 (89%)	7 (70%)
Dominant side measured	17 (94%)	9 (90%)
Number of days between measurement occasions ^b^	6 (1–8)	6 (1–7)
Difference in time of day between measurement occasions (hours) ^b^	0.5 (0–4.0)	1.0 (0–1.8)

^*a*^*presented in mean (SD),* ^*b*^ presented in median (range), * statistical difference between age groups: p < 0.05

The learning curves (Additional file 2), revealed that the SEM of the second set of older adults (*n* = 6) was less than half the SEM of the first set of older adults (*n =* 6) for the CSA of FDB, thickness of QP, AbDM, PF_prox_, PF_dist_, and FHL_long_, whereas the SEM was stable (i.e., the learning curve flattened) between the second and the third set of participants. Therefore, for these morphology measures, the data of the first set of older adults was omitted from the reliability analysis. This resulted in the final SEM to be 18 to 50% lower compared to when no data was omitted (Additional file 3). The SEM remained within the critical limits over the three sets of participants for all other morphology measures. Therefore, for these remaining morphology measures, the data of all participants were included in the analysis. The first set of participants (*n* = 6) from which data was omitted was not statistically different from the remaining participants (*n* = 12) on the demographics. The groups’ mean morphology measures for each of the two occasions are listed in Table 2.

**Table 2:** Descriptive statistics for the repeated measurements for ultrasound morphology of selected foot muscles and plantar fascia

		Older					Younger									
		**Tablet-based**					**Tablet-based**					**Mainframe**				
		n	Occ 1		Occ 2		n	Occ 1		Occ 2		n	Occ 1		Occ 2	
			Mean	SD	Mean	SD		Mean	SD	Mean	SD		Mean	SD	Mean	SD
AbH	CSA	18	198	(52)	201	(50)	10	194	(65)	189	(65)	10	192	(65)	191	(71)
	Th	18	10.3	(2.0)	10.3	(2.0)	10	10.9	(2.3)	10.8	(2.4)	10	10.9	(2.3)	10.6	(2.2)
FDB	CSA	12	248.5	(66.1)	247.8	(80.1)	10	211	(50)	209	(51)	10	210	(56)	209	(52)
	Th	18	10.4	(2.0)	10.2	(2.2)	10	10.9	(2.5)	10.4	(1.9)	10	11.0	(2.6)	10.4	(2.0)
QP	CSA	18	176	(35)	177	(34)	0	–	–	–	–	0	–	–	–	–
	Th	12	8.8	(1.3)	9.1	(1.4)	10	10.0	(1.0)	9.8	(1.1)	10	9.9	(1.0)	9.9	(1.0)
FHB	Th	18	13.6	(1.7)	13.7	(1.6)	10	14.4	(2.3)	14.1	(1.9)	10*	14.9	(2.1)	15.3	(1.7)
AbDM	Th	12	8.9	(1.4)	9.4	(1.5)	10	11.1	(1.8)	11.1	(2.2)	10	11.0	(2.1)	11.3	(2.1)
PF_prox_	Th	12	3.9	(0.8)	4.0	(1.0)	10	3.5	(0.6)	3.5	(0.7)	10*	3.3	(0.6)	3.2	(0.7)
PF_mid_	Th	18	2.3	(0.4)	2.4	(0.3)	10	2.1	(0.4)	2.2	(0.3)	10*	2.1	(0.4)	2.0	(0.4)
PF_dist_	Th	12	1.3	(0.2)	1.4	(0.2)	10	1.2	(0.2)	1.2	(0.2)	10	1.2	(0.2)	1.1	(0.2)
TA_long_	Th	18	25.2	(3.6)	25.3	(3.2)	10	22.4	(3.0)	22.7	(1.6)	10	22.6	(2.9)	22	(1.7)
TA_trans_	Th	18	24.4	(3.2)	24.6	(3.2)	10	22.0	(2.9)	21.8	(1.2)	10	22.4	(2.7)	21.7	(1.4)
FDL	CSA	18	191	(47)	197	(55)	10	154	(64)	138	(58)	10	146	(60)	142	(60)
PER_long_	Th	18	14.1	(2.2)	14.5	(2.1)	10	13.6	(1.9)	13.2	(2.3)	10	14.3	(2.2)	13.6	(2.7)
PER_trans_	Th	18	13.4	(2.3)	14.2	(2.2)	10	13.9	(2.0)	13.5	(2.6)	10	13.6	(2.3)	13.1	(2.8)
FHL_long_	Th	12	25.1	(3.5)	25.3	(3.1)	8	24.4	(2.6)	24.2	(2.6)	8	23.8	(3.6)	24.6	(3.4)
FHL_trans_	Th	7	24.8	(4.5)	24.8	(4.1)	10	22.7	(3.5)	22.4	(3.2)	10	22.7	(3.8)	22.5	(4.0)

*Occ: occasion, CSA: cross-sectional area, th: thickness, AbH: m. abductor hallucis, FDB: m. flexor digitorum brevis, QP: m. quadratus plantae, FHB: m. flexor hallucis brevis, AbDM: m. abductor digiti minimi, PF: plantar fascia, prox: proximal, mid: middle, dist: distal, TA: m. tibialis anterior, long: longitudinal, trans: transverse, FDL: m. flexor digitorum longus, PER: m. musculus peroneus, FHL: m. flexor hallucis longus. Values are presented in mm (thickness) and mm*^*2*^ (cross-sectional area). * indicates a statistical difference of ultrasound measures across machines: p < 0.05

**Fig. 3:**
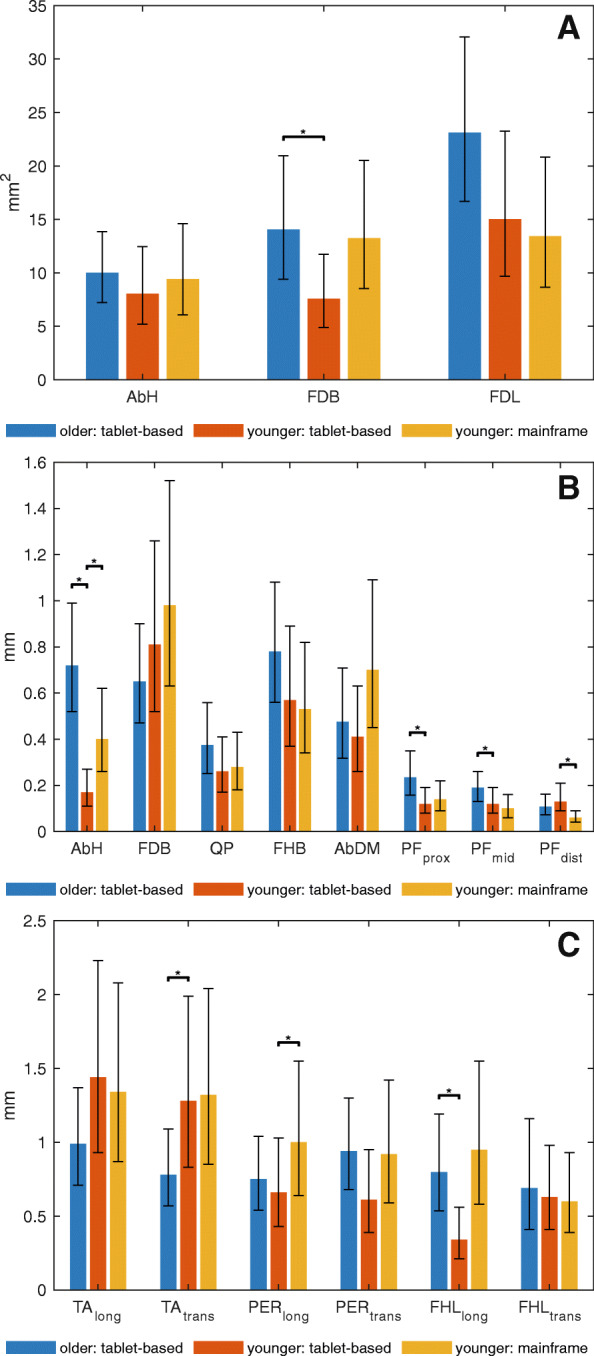
**A-C** Standard error of measurement (SEM) for cross-sectional area (2A) and thickness of intrinsic foot muscles and plantar fascia (2B) and extrinsic foot muscles (2C). * indicates a significant difference (*P* < 0.05) across age groups or ultrasound machines. AbH: m. abductor hallucis, FDB: m. flexor digitorum brevis, FDL: m. flexor digitorum longus, QP: m. quadratus plantae, FHB: m. flexor hallucis brevis, AbDM: m. abductor digiti minimi, PF: plantar fascia, prox: proximal, mid: middle, dist: distal, TA: m. tibialis anterior, long: longitudinal, trans: transverse, PER: m. peroneus, FHL: m. flexor hallucis longus

Table 3 and Fig. 3A-C show the intra-assessor measurement properties (i.e., ICC, SEM and SDC) for each muscle and morphology measure (i.e., CSA and thickness). The exact *p*-values for the comparison of the error variances, are listed in Additional file 3. The raw data on which the measurement properties are based are graphically presented in Additional file 4.

**Table 3:** Intra-assessor measurement properties for ultrasound morphology of selected foot muscles and plantar fascia

		Older								Younger															
		**Tablet-based**								**Tablet-based**								**Mainframe**							
		n	ICC	CI -	CI +	SEM	%SEM	SDC	%SDC	n	ICC	CI -	CI +	SEM	%SEM	SDC	%SDC	n	ICC	CI -	CI +	SEM	%SEM	SDC	%SDC
AbH	CSA	18	0.96	0.90	0.99	10	5.0	28	13.9	10	0.98	0.93	1.00	8	4.2	22	11.6	10	0.98	0.93	1.00	9	4.9	26	13.6
	Th	18	0.87	0.68	0.95	0.7*	7.0	2.0	19.3	10	0.99	0.98	1.00	0.2	1.6	0.5	4.5	10	0.97	0.84	0.99	0.4*	3.7	1.1	10.3
FDB	CSA	12	0.96	0.88	0.99	14*	5.7	39	15.7	10	0.98	0.91	0.99	8	3.6	21	10.0	10	0.94	0.77	0.99	13	6.3	37	17.6
	Th	18	0.91	0.77	0.96	0.7	6.3	1.8	17.4	10	0.87	0.58	0.97	0.8	7.6	2.3	21.0	10	0.82	0.47	0.95	1.0	9.2	2.7	25.4
QP	CSA	18^a^	0.75	0.44	0.90	17	9.7	47	26.8	0	–	-	-	–	–	–	–	0	–	-	-	–	–	–	–
	Th	12	0.92	0.70	0.98	0.4	4.2	1.0	11.6	10	0.93	0.77	0.98	0.3	2.6	0.7	7.3	10	0.93	0.73	0.98	0.3	2.8	0.8	7.8
FHB	Th	18	0.78	0.50	0.91	0.8	5.7	2.2	15.7	10	0.93	0.75	0.98	0.6	4.0	1.6	11.2	10	0.93	0.74	0.98	0.5	3.5	1.5	9.7
AbDM	Th	12	0.89	0.41	0.97	0.5	5.2	1.3	14.3	10^a^	0.96	0.85	0.99	0.4	3.7	1.1	10.2	10^a^	0.89	0.64	0.97	0.7	6.3	2.0	17.4
PF_prox_	Th	12^b^	0.94	0.80	0.98	0.23*	6.0	0.65	16.5	10	0.96	0.87	0.99	0.12	3.5	0.34	9.6	10	0.95	0.80	0.99	0.14	4.3	0.40	12.0
PF_mid_	Th	18	0.70	0.35	0.88	0.19*	7.7	0.51	21.4	10	0.88	0.60	0.97	0.12	5.8	0.34	16.0	10	0.93	0.75	0.98	0.10	4.8	0.28	13.2
PF_dist_	Th	12	0.57	0.06	0.85	0.11	8.0	0.30	22.2	10	0.44	−0.18	0.82	0.13	11.0	0.37	30.6	10	0.89	0.65	0.97	0.06*	5.0	0.15	13.9
TA_long_	Th	18^a^	0.92	0.79	0.97	1.0	3.9	2.7	10.8	10	0.62	0.02	0.89	1.4	6.4	4.0	17.7	10	0.68	0.17	0.91	1.3	6.0	3.7	16.7
TA_trans_	Th	18	0.94	0.85	0.98	0.8*	3.2	2.2	8.9	10	0.67	0.08	0.91	1.3	5.9	3.6	16.2	10	0.63	0.09	0.89	1.3	6.0	3.7	16.6
FDL	CSA	18	0.79	0.53	0.92	23	11.9	64	33.1	10	0.94	0.39	0.99	15	10.3	42	28.5	10	0.95	0.82	0.99	13	9.3	37	25.9
PER_long_	Th	18	0.88	0.69	0.95	0.8	5.3	2.1	14.6	10	0.90	0.67	0.97	0.7	5.0	1.8	13.7	10	0.84	0.49	0.96	1.0*	7.2	2.8	19.9
PER_trans_	Th	18	0.84	0.43	0.95	0.9	6.8	2.6	18.9	10	0.93	0.76	0.98	0.6	4.5	1.7	12.4	10	0.87	0.59	0.97	0.9	6.9	2.6	19.0
FHL_long_	Th	12	0.94	0.81	0.98	0.8*	3.2	2.2	8.8	8	0.98	0.92	1.00	0.3	1.4	1.0	3.9	8	0.93	0.61	0.99	1.0	3.9	2.6	10.8
FHL_trans_	Th	7	0.97	0.86	1.00	0.7	2.8	1.9	7.7	10	0.96	0.87	0.99	0.6	2.8	1.8	7.7	10	0.98	0.91	0.99	0.6	2.7	1.7	7.3

ICC: intra-class correlation coefficient, CI- and CI+: lower and upper limit of the 95% confidence interval, SEM: standard error of measurement, SDC: smallest detectable change, CSA: cross-sectional area, th: thickness, AbH: m. abductor hallucis, FDB: m. flexor digitorum brevis, QP: m. quadratus plantae, FHB: m. flexor hallucis brevis, AbDM: m. abductor digiti minimi, PF: plantar fascia, prox: proximal, mid: middle, dist: distal, TA: m. tibialis anterior, long: longitudinal, trans: transverse, FDL: m. flexor digitorum longus, PER: m. musculus peroneus longus + brevis, FHL: m. flexor hallucis longus. The units of measurement for the SEM and SDC are mm (thickness), mm^2^ (CSA), or a percentage of the group mean muscle size (%SEM and %SDC). ^a^ indicates a non-normal distribution for the difference between the repeated measurements. ^b^ indicates the presence of heteroscedasticity. * indicates a significant difference (*P <* 0.05) across age groups or ultrasound machines

Table 3 demonstrates that, in older adults, the ICC of intrinsic foot muscle and PF morphology measures ranged from 0.57 (PF_dist_) to 0.96 (CSA AbH and FDB). In the older adult group, the SEM did not exceed an absolute value of 1.0 mm for the thickness of any of the foot muscles or the PF. Relative to the average tissue size, in older adults, the SEM was smallest for the thickness of FHL (2.8 and 3.2%) and TA (3.2 and 3.9%) and largest for the CSA of FDL (11.9%), followed by the CSA of QP (9.7%). Considering only the thickness measures in the older adult group, the largest relative SEM was found for PF_mid_ (7.7%) and PF_dist_ (8.0%). When the relative SEM in older adults was compared between the two morphology measures for the same muscle, smaller values were observed for the CSA of both the AbH and FDB (AbH: 5.0% vs. 7.0%; FDB: 5.7% vs. 6.3%).

Comparing the age groups (Table 3, Fig. 3A-C), it was shown that the SEM was significantly greater in older adults for the thickness of AbH, CSA of FDB, PF_prox_, PF_mid_ and FHL_long_. The corresponding relative SEM ranged from 3.2% (FHL_long_) to 7.7% (PF_mid_) in older adults versus 1.4% (FHL_long_) to 5.8% (PF_mid_) in younger adults. In contrast, the thickness of TA_trans_ was measured with a significantly smaller measurement error and more reliably in older adults compared to younger adults (SEM: 0.8 mm (3.2%) vs. 1.3 mm (5.9%), ICC: 0.94 vs. 0.67).

The comparison between the two ultrasound machines (Table 3, Fig. 3A-C) revealed a statistically larger SEM for the thickness of AbH and PER_long_ when the mainframe machine was used compared to when the tablet-based machine was used (AbH: 0.4 mm (3.7%) vs. 0.2 mm (1.6%); PER_long_: 1.0 mm (7.2%) vs. 0.7 mm (5.0%)). For PF_dist_, a significantly smaller SEM was achieved when the images were obtained with the mainframe machine (0.06 mm (5.0%) vs 0.13 mm (11.0%)).

## Discussion

In this study, we assessed the intra-assessor reliability and measurement error for the morphology of selected foot muscles and PF derived from ultrasound images collected by a single operator using a tablet-based machine in older adults. We also compared the measurement error with that obtained in younger adults and examined the influence of the ultrasound machine that was used. The results showed that the morphology of most of the assessed muscles can be reliably assessed with acceptable measurement error in older adults when using a tablet-based device, although measurement errors were smaller for some muscles in younger adults. In general, using a mainframe machine did not improve the repeatability in younger adults. In fact, when the repeatability differed between the machines, the repeatability of muscle morphology measures was superior for the tablet-based machine.

The morphology of foot muscles could be assessed in older adults with an error ranging from 2.8 to 11.9%, equating to an SDC of 7.7 to 33.1%. When omitting the CSA of FDL, which showed an exceptionally large error, and selecting the most accurate morphology measure (i.e., CSA or thickness) for each muscle, the SDC was 15.7% at its greatest extent in the older adult group using the table-based device. This means that, on an individual level, a change in foot muscle size beyond 15.7% can be considered a real change [38]. In order to be a meaningful metric to measure a group mean change in muscle morphology, for example in a prospective intervention study, this change should exceed the SDC divided by the square root of the sample size [50]. A group change of this magnitude is realistic as an 8-week foot strengthening intervention in younger adults showed average foot muscle hypertrophy ranging from 5 to 15% [17] and, in general, older adults are expected to have a similar response to strength training [51]. Whether the morphological changes of foot muscles as a response to training in older adults indeed exceed the SDCs, needs to be investigated in future studies. Our range of SDCs corroborates well with the limits of agreement (LoA), a metric comparable with the SDC [52], reported by previous studies where the same muscles were examined in younger populations by operators with 8 years of ultrasound experience using more advanced machines [33, 36]. Next to the measurement errors, the reliability of the muscle morphology measures in older adults was predominantly ‘excellent’ and at least ‘good’ (ICC: 0.75 to 0.96). This signifies that overall the measurement error is small enough relative to the between-subject variance [38], enabling us to differentiate between older individuals.

Considering direct measures for toe flexor strength, previous research [21] revealed poorer reliability for toe flexor strength measures compared to ultrasound morphology (i.e., an indirect strength measure). In addition, a study in older adults [53] indicated a larger measurement error for toe flexor strength measurements than reported in the current study for foot muscle morphology. Together with the favorable ability of ultrasound to assess individual muscles, and although being an indirect strength measure, this supports its use to study the role of foot muscles in the older adult population. Future studies can use this methodology to investigate the effect of a foot strengthening program on foot muscle morphology in older adults. The latter also meets the requirement for a prospective study to approve the responsiveness of ultrasound to detect foot muscle hypertrophy in older adults.

For AbH and FDB, the cross-sectional area was measured with a slightly smaller error when compared to the thickness of the same muscle in older adults. The human error is assumed to affect the straightforward linear distance between the deeper and superficial epimysium (i.e., thickness) to a lesser degree than the demarcation of a muscle’s outline (i.e., CSA) [37]. Apparently, this advantage did not outweigh the larger image capturing variability associated with longitudinal imaging of these muscles with an oval-like cross-section. The superior repeatability for the CSA of AbH and FDB is auspicious as a two-dimensional quantity is better able to cover a non-uniform change in muscle morphology that may occur in response to exercise or muscle disuse [28]. The SDC for AbH’s CSA in the current study was substantially smaller than what was found using MRI (28 vs. 46.1 mm^2^) and comparable for the CSA of FDB (39 vs. 36.4 mm^2^) [54]. The ability to accurately measure the CSA of AbH and FDB in older adults is further promising as AbH and FDB are, together with QP, the intrinsic foot muscles most closely aligned with the medial longitudinal arch of the foot. Hence, these are key structures in the investigation of foot function [55, 56].

 In contrast to FDB and AbH, QP is a deeper located muscle and is, therefore, less accessible by ultrasound [57]. Indeed, the results show that QP’s CSA was measured with only small precision in older adults, the SDC being 26.8%. This SDC is three times worse than shown in a previous study in younger adults [33]. Apart from the contrasting study populations, the studies differ substantially at the level of experience of the operators (i.e., 10 months vs. 8 years) and the ultrasound machines used (tablet-based vs. mainframe). The post-processing delineating of the QP’s fascial borders, was experienced as extremely difficult by the researcher of the current study. Partially, this was due to the oblique orientation of the tissues adjacent to the medial aspect of the QP (e.g., plantar nerves, FDL tendon). This causes the ultrasound beam to reflect away from these tissues [57], resulting in an isoechoic appearance of QP and its surroundings. Together with the poor lateral resolution at this depth, this may have led to inaccuracy in the definition of the muscle’s envelope. In contrast to the SEM for the CSA, the SEM for the thickness of QP was reasonably low and presents, therefore, a better alternative to quantify the morphology of QP for operators with similar experience.

Three muscle morphology measures (CSA: FDB; thickness: AbH, FHL_long_) showed superior repeatability in younger adults compared to older adults. This may be caused by an age-related decline in muscle quality, such as a higher degree of intramuscular adipose tissue [39], which indeed has been observed in the PIFMs [58]. As a consequence, the ultrasound beam scatters and sound is largely absorbed before reaching the deeper epimysial border, preventing it to appear as a bright hyperechoic structure [57]. Further, muscle contractions, aimed to aid the offline muscle delineation, were not always accomplished in the older adults as they tended to fall asleep or the researcher observed difficulties to relax the muscle after contraction. Although the superior repeatability for some muscle morphology quantifications in the younger age group was in line with our expectations, the measurement errors for these morphology measures were extremely low in the younger adults (SEM: 1.4–3.6%) and acceptable in older adults (SEM: 3.7–7.0%). Surprisingly, TA_trans_ showed less measurement error in older adults (SEM: 3.2 vs. 5.9%) which could be explained by its more consistent shape in longitudinal direction compared to in younger adults.

Against our expectations, for the muscles showing dissimilar repeatability across the machines (i.e., thickness AbH and PER_long_), the tablet-based device turned out to be advantageous. Apparently, for these muscles, the larger field of view owing to the larger footprint of the probe of the mainframe system and its expected superior image quality due to the use of advanced options were of insufficient benefit. The tabled-based device, instead, is equipped with a smaller probe and lighter cables, minimizing the chance for unintentional transducer manipulation that would be at the cost of the perpendicularity of the image captured and thus repeatability [57]. The similar performance across the machines indicates that for each muscle the image quality of the tablet-based machine was sufficient to delineate the epimysial borders, which has previously been demonstrated for the thickness and CSA of AbH using a laptop-based machine [37]. Likewise, another study observed invariant thickness of hip extensor muscles across two different ultrasound machines [59]. Our findings confirm the assumption from other authors that machines can be used interchangeably in the assessment of muscle morphology [41], except for FHB, which showed a systematic difference across the machines in our study. Hence, this implies that health care professionals are not contingent on the availability of a specific machine to monitor muscle morphological changes over time. It further indicates that measurements for foot muscles morphology are not restricted to well-equipped hospitals or research labs, but can easily take place at the home of aged or diseased populations. This is promising given the current implementation of transitioning care from hospital to peoples own home.

The relative SDCs for PF, a passive structure associated with foot posture [19], increased the more distal it was assessed, ranging from 16.5 to 22.2% in older adults. In younger adults the SDC was significantly smaller for the proximal and middle portion (PF_prox_: 9.6%; PF_mid_: 16.0%), resembling previously reported inter-assessor repeatability [34]. Callus may have interfered with image quality [43] in older adults more than in younger as this skin condition is more prevalent in that population [40] and especially manifested in the region of PF_prox_ and PF_mid_. Changing to the mainframe machine improved the repeatability for the distal portion of PF to an SDC of 13.9% and is, as such, also in agreement with the literature [34]. This can be explained by the enhanced visibility of deeper tissues (e.g., 2nd metatarsal bone) through the advanced penetration of sound by the mainframe machine, aiding the correct probe position [30]. Whenever available, a mainframe machine is, therefore, recommended to measure the thickness of PF in the feet of older adults when callus is present.

The current study is the first to investigate the reliability and measurement error for a large selection of both intrinsic and extrinsic foot muscles, in addition to PF, in older adults. Accounting for the operator’s proficiency and excluding measurements accordingly ensured a valid comparison of the measurement properties between older and younger adults. Nevertheless, the study was subjected to several limitations that need to be considered. Most importantly, this intra-assessor reliability study does not provide information on the validity of the ultrasound morphology measures. Although the operator was a novice scanner at the start of the training, she received intensive specific training in scanning the foot muscles according to a fixed protocol. In addition, detailed anatomical knowledge of the scanner and the triangulation during the training program further contributed to valid ultrasound measurements. Nevertheless, because of the uncertain accuracy of the morphology measures, a systematic error cannot be ruled out. A systematic error would influence the mean morphology measure itself and, as a consequence, the relative measurement properties (i.e., relative SEM and SDC), but not the absolute measurement properties. However, it is not expected that this has occurred substantially. In addition, the repeated measurements were sometimes only one or two days apart. Therefore, the image capturing at the second occasion may have been subjected to recall bias. Nevertheless, this bias is expected to be marginal, considering the straightforward scan protocol. Further, the measurement properties were estimated from samples consisting of 10 to 18 participants, believed to be relatively small [60]. However, the sample size of the older adult group exceeded that of other ultrasound reliability studies with this amount of tissues [33, 34]. Another limitation is that the comparison between machines only pertains to younger adults, as we decided to extend the protocol after the data collection was completed in older adults. Whilst it is unclear how a change of machine affects the repeatability in older adults, the measurement properties for the tablet-based device were already promising for its future use. The results are, however, limited to a subset of tissue morphology measures determined from pilot testing with the tablet-based device. Hence, the applicability of the mainframe machine in the assessment of, in particular, the CSA of muscles such as QP, FHB and AbDM remains elusive. Lastly, only a single operator was involved in this study which means that the measurement properties cannot simply be generalized to any other operator [60]. This is because not only is the quality and consistency of the acquired images determined by the ultrasound experience and the background of the operator, but also does the post-processing delineation or identification of muscle borders rely heavily on the anatomical knowledge of the rater [61]. Nevertheless, a 10-month period of intense specific training, during which a specific foot muscle scan protocol was used, appeared to be sufficient to obtain good to excellent reliability and measurement error, but only when a single operator performs the measurements.

## Conclusion

The results of this intra-assessor reliability study showed that a tablet-based ultrasound machine can be reliably used to assess the morphology of selected foot muscles in older adults, with the exception of plantar fascia thickness. This supports the use of this instrument in future studies to gain understanding of the role of these foot muscles in foot function. Although the measurement errors were smaller in younger adults for some muscle morphology measures, they seem adequate in older adults to detect hypertrophy as a response to training on a group level. The use of a tablet-based device seems to be a good alternative to a mainframe system, but how its superior repeatability applies to older adults needs to be further investigated. Nevertheless, our findings advocate the use of ultrasound in future studies or in clinical practice when foot muscle morphology is the outcome of interest, without being restricted to expensive ultrasound machines that often have limited access. In addition, the use of a tablet-based device enables the researcher or clinician to perform the ultrasound measurements at any location, even at the home of the older adults.

## Supplementary Information

**Additional file 1.** Protocol for imaging the selected foot muscles and plantar fascia.

**Additional file 2.** Learning curves: standard error of measurement for consecutive sets of participating older adults.

**Additional file 3.** Intra-assessor measurement properties with corresponding confidence intervals and exact *p*-values for comparisons.

**Additional file 4.** Graphical presentation of the raw data on which the measurement properties are based.

## Data Availability

The dataset supporting the conclusions of this article is available on request in the DataverseNL repository, 10.34894/XNLROB .

## Abbreviations

PIFM: plantar intrinsic foot muscles
MRI: magnetic resonance imaging
CSA: cross-sectional area
ICC: intra-class correlation coefficient
CI: Confidence interval
TA: tibialis anterior muscle
PER: peroneus muscle
FDL: flexor digitorum longus muscle
AbH: abductor hallucis muscle
FHL: flexor hallucis longus muscle
FDB: flexor digitorum brevis muscle
QP: quadratus plantae muscle
FHB: flexor hallucis brevis muscle
AbDM: abductor digiti minimi muscle
PF: plantar fascia muscle
SEM: standard error of measurement
SDC: smallest detectable change
Prox: proximal portion
Mid: middle portion
Dist: distal portion
Long: longitudinal
Trans: transverse
LoA: limits of agreement
