# Supplementary material for: Intra-assessor reliability and measurement error of ultrasound measures for foot muscle morphology in older adults using a tablet-based ultrasound machine
Source: J Foot Ankle Res. 2022 Jan 25;15:6. doi: 10.1186/s13047-022-00510-1 (PMC8788121; doi:10.1186/s13047-022-00510-1)
Supplement: Supplementary file 1 — Additional file 1. Protocol for imaging the selected foot muscles and plantar fascia. [file 13047_2022_510_MOESM1_ESM.docx]

Protocol for imaging the foot tissues, which is based on previous research (1,2) and our own pilot testing.

| The **extrinsic foot muscles** were first imaged on the short axis in the extension of the drawing line and then on the long axis by rotating the probe 90°. | | |
| --- | --- | --- |
| **Location** | **Transverse plane** | **Longitudinal plane** |
| **TA** was imaged at 25% of LLLL, lateral to the anterior border of the tibia. | The interosseous membrane was kept as horizontal as achievable. | Attempts were made to image the aponeurosis in the middle of the muscle belly. |
| **PER** was imaged at 50 % of LLLL, such that the muscle was imaged on top of the fibula. |  |  |
| **FDL** was imaged on the short axis only at 50 % of MLLL | The position and orientation of the probe was adjusted in a way the muscle was least obscured by the tibia. |  |
| **FHL** was imaged at 80 % of MLLL. |  | The myotendinous junction of the soleus muscle could often be observed. |
| The **intrinsic foot muscles** were first imaged on the long axis and then on the short axis. | | |
| **AbH** | The scan was made on a line directly anterior to the medial malleolus and perpendicular to the long axis of the foot. | The probe was placed at the muscle’s origin on the medial process of the calcaneal tuberosity, moving towards just inferior to the navicular tuberosity until the thickest part of the muscle belly was captured. |
| **FDB** | At the thickest point, the probe was rotated 90° for the transverse image. | A scan was made on the drawing between the medial process of the calcaneal tuberosity and the third ray where the muscle showed its thickest part. |
| **QP** | At the QP’s thickest portion, the probe was rotated 90° to capture the muscle’s cross section. | The spring ligament was used as an internal landmark and located by placing the probe at the medial plantar side of the foot. |
| **FHB** |  | The probe was placed along the shaft of the first metatarsal bone on a line joining the medial process of the calcaneal tuberosity and the first metatarsophalangeal joint. |
| **AbDM** |  | An image was captured where the muscle showed its thickest part on a line between its origin to the lateral tuberosity and the fifth metatarsal head. |
| The **PF** was imaged at three locations on a line between its attachment to the medial process of the calcaneal tuberosity and the second ray. |  | The proximal fascia (PF_prox_) was imaged distal to its attachment to the calcaneus, where the visibility of the PF was not obstructed by anisotropy. Moving the probe along the scan line, the middle fascia (PF_mid_) was located halfway its slope adjacent to the fat path and the distal fascia (PF_dist_) halfway the base and the head of the second metatarsal bone. |

*MLLL: medial lower leg length - the distance between the fibular head and the inferior border of the lateral malleolus. LLLL: laterally lower leg length - the distance between the medial knee joint and the inferior border of the medial malleolus.*

1. Mickle KJ, Nester CJ, Crofts G, Steele JR. Reliability of ultrasound to measure morphology of the toe flexor muscles. J Foot Ankle Res. 2013/04/06. 2013;6(1):12.

2. Crofts G, Angin S, Mickle KJ, Hill S, Nester CJ. Reliability of ultrasound for measurement of selected foot structures. Gait Posture. 2013/06/25. 2014;39(1):35–9.
