# Supplementary material for: Intra-assessor reliability and measurement error of ultrasound measures for foot muscle morphology in older adults using a tablet-based ultrasound machine
Source: J Foot Ankle Res. 2022 Jan 25;15:6. doi: 10.1186/s13047-022-00510-1 (PMC8788121; doi:10.1186/s13047-022-00510-1)
Supplement: Supplementary file 2 — Additional file 2. Learning curves: standard error of measurement for consecutive sets of participating older adults. [file 13047_2022_510_MOESM2_ESM.docx]

#
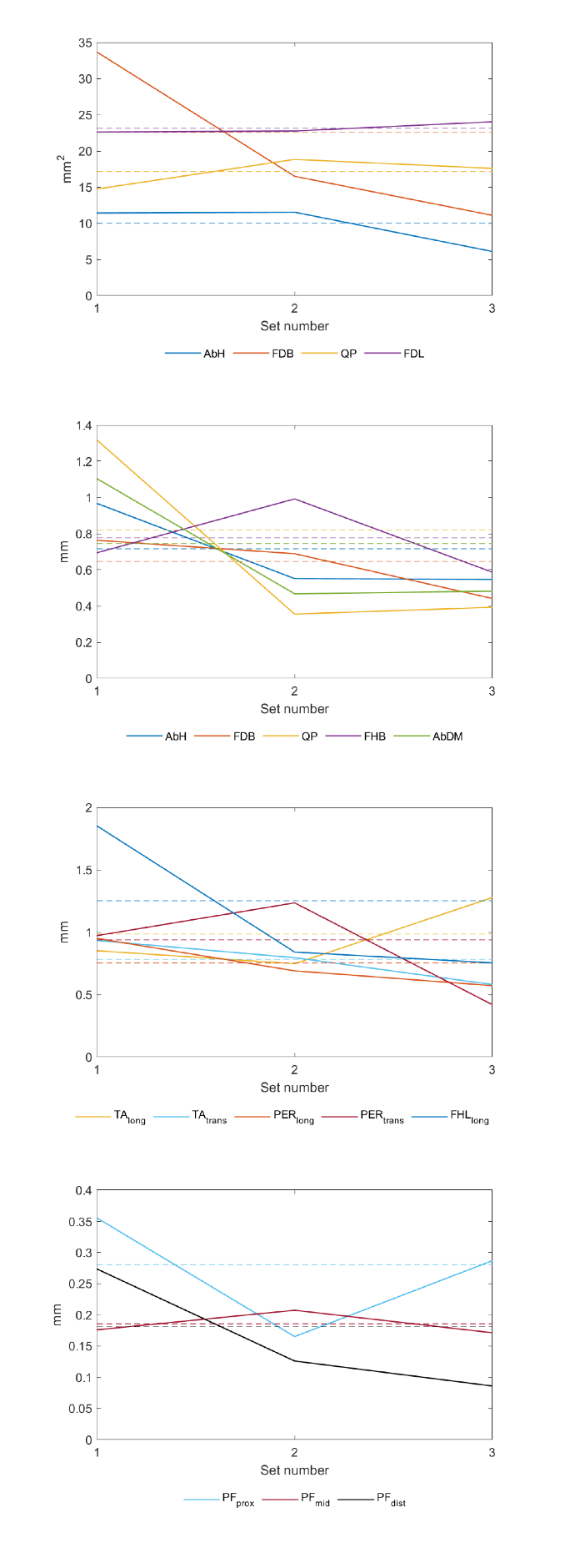


***Learning curves: standard error of measurement for consecutive sets of participating older adults****. AbH: m. abductor hallucis, FDB: m. flexor digitorum brevis, QP: m. quadratus plantae, FHB: m. flexor hallucis brevis, AbDM: m. abductor digiti minimi, PF: plantar fascia, prox: proximal, mid: middle, dist: distal, TA: m. tibialis anterior, long: longitudinal, trans: transversal, FDL: m. flexor digitorum longus, PER: m. musculus peroneus, FHL: m. flexor hallucis longus. Dotted lines indicate the standard error of measurement when all participants are included.*
