# Supplementary material for: Intra-assessor reliability and measurement error of ultrasound measures for foot muscle morphology in older adults using a tablet-based ultrasound machine
Source: J Foot Ankle Res. 2022 Jan 25;15:6. doi: 10.1186/s13047-022-00510-1 (PMC8788121; doi:10.1186/s13047-022-00510-1)
Supplement: Supplementary file 3 — Additional file 3. Intra-assessor measurement properties with corresponding confidence intervals and exact p-values for comparisons. [file 13047_2022_510_MOESM3_ESM.docx]

# ***Table 4****. intra-assessor measurement properties for ultrasound morphology of selected foot muscles and plantar fascia in older adults.*

|  |  | n | ICC | CI - | CI + | SEM | p | CI - | CI + | %SEM | SDC | CI - | CI + | %SDC |
| --- | --- | --- | --- | --- | --- | --- | --- | --- | --- | --- | --- | --- | --- | --- |
| AbH | CSA | 18 | 0.96 | 0.90 | 0.99 | 10 | 0.2587 | 7 | 14 | 5.0 | 28 | 20 | 38 | 13.9 |
|  | Th | 18 | 0.87 | 0.68 | 0.95 | 0.7 | 0.0001 | 0.5 | 1.0 | 7.0 | 2.0 | 1.4 | 2.8 | 19.3 |
| FDB | CSA | 18 | 0.88 | 0.70 | 0.95 | 23 | 0.0011 | 16 | 31 | 9.4 | 63 | 45 | 87 | 26.0 |
|  |  | 12 | 0.96 | 0.88 | 0.99 | 14.1 | 0.0368 | 9.4 | 21.0 | 5.7 | 39.0 | 26.1 | 58.1 | 15.7 |
|  | Th | 18 | 0.91 | 0.77 | 0.96 | 0.7 | 0.1995 | 0.5 | 0.9 | 6.3 | 1.8 | 1.3 | 2.5 | 17.4 |
| QP | CSA | 18^a^ | 0.75 | 0.44 | 0.90 | 17 | 0.5241 | 12 | 24 | 9.7 | 47 | 34 | 66 | 26.8 |
|  | Th | 18 | 0.60 | 0.19 | 0.83 | 0.8 | 0.0007 | 0.6 | 1.1 | 9.2 | 2.3 | 1.6 | 3.2 | 25.5 |
|  |  | 12 | 0.92 | 0.70 | 0.98 | 0.4 | 0.1464 | 0.3 | 0.6 | 4.2 | 1.0 | 0.7 | 1.5 | 11.6 |
| FHB | Th | 18 | 0.78 | 0.50 | 0.91 | 0.8 | 0.1752 | 0.6 | 1.1 | 5.7 | 2.2 | 1.6 | 3.0 | 15.7 |
| AbDM | Th | 18^a^ | 0.76 | 0.45 | 0.90 | 0.8 | 0.0346 | 0.5 | 1.0 | 8.1 | 2.1 | 1.5 | 2.9 | 22.5 |
|  |  | 12 | 0.89 | 0.41 | 0.97 | 0.5 | 0.3267 | 0.3 | 0.7 | 5.2 | 1.3 | 0.9 | 2.0 | 14.3 |
| PF_prox_ | Th | 18^a^ | 0.87 | 0.69 | 0.95 | 0.28 | 0.0071 | 0.20 | 0.39 | 7.2 | 0.78 | 0.56 | 1.08 | 19.9 |
|  |  | 12^b^ | 0.94 | 0.80 | 0.98 | 0.23 | 0.0284 | 0.2 | 0.3 | 6.0 | 0.65 | 0.43 | 0.97 | 16.5 |
| PF_mid_ | Th | 18 | 0.70 | 0.35 | 0.88 | 0.19 | 0.0988 | 0.13 | 0.26 | 7.7 | 0.51 | 0.37 | 0.71 | 21.4 |
| PF_dist_ | Th | 18 | 0.44 | 0.01 | 0.74 | 0.18 | 0.1706 | 0.13 | 0.25 | 13.9 | 0.50 | 0.36 | 0.70 | 38.6 |
|  |  | 12 | 0.57 | 0.06 | 0.85 | 0.11 | 0.2607 | 0.1 | 0.2 | 8.0 | 0.30 | 0.20 | 0.45 | 22.2 |
| TA_long_ | Th | 18^a^ | 0.92 | 0.79 | 0.97 | 1.0 | 0.0851 | 0.7 | 1.4 | 3.9 | 2.7 | 2.0 | 3.8 | 10.8 |
| TA_trans_ | Th | 18 | 0.94 | 0.85 | 0.98 | 0.8 | 0.0383 | 0.6 | 1.1 | 3.2 | 2.2 | 1.6 | 3.0 | 8.9 |
| FDL | Th | 18 | 0.79 | 0.53 | 0.92 | 23 | 0.0942 | 17 | 32 | 11.9 | 64 | 46 | 89 | 33.1 |
| PER_long_ | Th | 18 | 0.88 | 0.69 | 0.95 | 0.8 | 0.3602 | 0.5 | 1.0 | 5.3 | 2.1 | 1.5 | 2.9 | 14.6 |
| PER_trans_ | Th | 18 | 0.84 | 0.43 | 0.95 | 0.9 | 0.0947 | 0.7 | 1.3 | 6.8 | 2.6 | 1.9 | 3.6 | 18.9 |
| FHL_long_ | Th | 18 | 0.81 | 0.57 | 0.92 | 1.3 | 0.0010 | 0.9 | 1.7 | 5.0 | 3.5 | 2.5 | 4.8 | 13.9 |
|  |  | 12 | 0.94 | 0.81 | 0.98 | 0.8 | 0.0173 | 0.5 | 1.2 | 3.2 | 2.2 | 1.5 | 3.3 | 8.8 |
| FHL_trans_ | Th | 7 | 0.97 | 0.86 | 1.00 | 0.7 | 0.3931 | 0.4 | 1.2 | 2.8 | 1.9 | 1.1 | 3.2 | 7.7 |

# *ICC: intra-class correlation coefficient, SEM: standard error of measurement, SDC: smallest detectable change, CI- and CI+: lower and upper limit of the 95% confidence interval, CSA: cross-sectional area, th: thickness, AbH: m. abductor hallucis, FDB: m. flexor digitorum brevis, QP: m. quadratus plantae, FHB: m. flexor hallucis brevis, AbDM: m. abductor digiti minimi, PF: plantar fascia, prox: proximal, mid: middle, dist: distal, TA: m. tibialis anterior, long: longitudinal, trans: transversal, FDL: m. flexor digitorum longus, PER: m. musculus peroneus, FHL: m. flexor hallucis longus. The units of measurement of the SEM and SDC are mm (thickness), mm^2^ (CSA), or a percentage of the group mean muscle size (%SEM and %SDC). ^a^ indicates a non-normal distribution of the difference between the repeated measurements. ^b^ indicates the presence of heteroscedasticity. p-value is presented for the comparison across age groups.*

# ***Table 5****. intra-assessor measurement properties for ultrasound morphology of selected foot muscles and plantar fascia in younger adults using a tablet-based machine.*

|  |  | n | ICC | CI - | CI + | SEM | CI - | CI + | %SEM | SDC | CI - | CI + | %SDC |
| --- | --- | --- | --- | --- | --- | --- | --- | --- | --- | --- | --- | --- | --- |
| AbH | CSA | 10 | 0.98 | 0.93 | 1.00 | 8 | 5 | 12 | 4.2 | 22 | 14 | 35 | 11.6 |
|  | Th | 10 | 0.99 | 0.98 | 1.00 | 0.2 | 0.1 | 0.3 | 1.6 | 0.5 | 0.3 | 0.8 | 4.5 |
| FDB | CSA | 10 | 0.98 | 0.91 | 0.99 | 8 | 5 | 12 | 3.6 | 21 | 14 | 33 | 10.0 |
|  | Th | 10 | 0.87 | 0.58 | 0.97 | 0.8 | 0.5 | 1.3 | 7.6 | 2.3 | 1.5 | 3.5 | 21.0 |
| QP | Th | 10 | 0.93 | 0.77 | 0.98 | 0.3 | 0.2 | 0.4 | 2.6 | 0.7 | 0.5 | 1.1 | 7.3 |
| FHB | Th | 10 | 0.93 | 0.75 | 0.98 | 0.6 | 0.4 | 0.9 | 4.0 | 1.6 | 1.0 | 2.5 | 11.2 |
| AbDM | Th | 10^a^ | 0.96 | 0.85 | 0.99 | 0.4 | 0.3 | 0.6 | 3.7 | 1.1 | 0.7 | 1.8 | 10.2 |
| PF_prox_ | Th | 10 | 0.96 | 0.87 | 0.99 | 0.12 | 0.08 | 0.19 | 3.5 | 0.34 | 0.22 | 0.52 | 9.6 |
| PF_mid_ | Th | 10 | 0.88 | 0.60 | 0.97 | 0.12 | 0.08 | 0.19 | 5.8 | 0.34 | 0.22 | 0.52 | 16.0 |
| PF_dist_ | Th | 10 | 0.44 | -0.18 | 0.82 | 0.13 | 0.09 | 0.21 | 11.0 | 0.37 | 0.24 | 0.57 | 30.6 |
| TA_long_ | Th | 10 | 0.62 | 0.02 | 0.89 | 1.4 | 0.9 | 2.2 | 6.4 | 4.0 | 2.6 | 6.2 | 17.7 |
| TA_trans_ | Th | 10 | 0.67 | 0.08 | 0.91 | 1.3 | 0.8 | 2.0 | 5.9 | 3.6 | 2.3 | 5.5 | 16.2 |
| FDL | Th | 10 | 0.94 | 0.39 | 0.99 | 15 | 10 | 23 | 10.3 | 42 | 27 | 65 | 28.5 |
| PER_long_ | Th | 10 | 0.90 | 0.67 | 0.97 | 0.7 | 0.4 | 1.0 | 5.0 | 1.8 | 1.2 | 2.9 | 13.7 |
| PER_trans_ | Th | 10 | 0.93 | 0.76 | 0.98 | 0.6 | 0.4 | 1.0 | 4.5 | 1.7 | 1.1 | 2.6 | 12.4 |
| FHL_long_ | Th | 8 | 0.98 | 0.92 | 1.00 | 0.3 | 0.2 | 0.6 | 1.4 | 1.0 | 0.6 | 1.6 | 3.9 |
| FHL_trans_ | Th | 10 | 0.96 | 0.87 | 0.99 | 0.6 | 0.4 | 1.0 | 2.8 | 1.8 | 1.1 | 2.7 | 7.7 |

# *ICC: intra-class correlation coefficient, SEM: standard error of measurement, SDC: smallest detectable change, CI- and CI+: lower and upper limit of the 95% confidence interval, CSA: cross-sectional area, th: thickness, AbH: m. abductor hallucis, FDB: m. flexor digitorum brevis, QP: m. quadratus plantae, FHB: m. flexor hallucis brevis, AbDM: m. abductor digiti minimi, PF: plantar fascia, prox: proximal, mid: middle, dist: distal, TA: m. tibialis anterior, long: longitudinal, trans: transversal, FDL: m. flexor digitorum longus, PER: m. musculus peroneus, FHL: m. flexor hallucis longus. The units of measurement of the SEM and SDC are mm (thickness), mm^2^ (CSA), or a percentage of the group mean muscle size (%SEM and %SDC). ^a^ indicates a non-normal distribution of the difference between the repeated measurements.*

# **Table 6**. *intra-assessor measurement properties for ultrasound morphology of selected foot muscles and plantar fascia in younger adults using a mainframe machine.*

|  |  | n | ICC | CI - | CI + | SEM | p | CI - | CI + | %SEM | SDC | CI - | CI + | %SDC |
| --- | --- | --- | --- | --- | --- | --- | --- | --- | --- | --- | --- | --- | --- | --- |
| AbH | CSA | 10 | 0.98 | 0.93 | 1.00 | 9 | 0.4039 | 6 | 15 | 4.9 | 26 | 17 | 40 | 13.6 |
|  | Th | 10 | 0.97 | 0.84 | 0.99 | 0.4 | 0.0363 | 0.3 | 0.6 | 3.7 | 1.1 | 0.7 | 1.7 | 10.3 |
| FDB | CSA | 10 | 0.94 | 0.77 | 0.99 | 13 | 0.0670 | 9 | 21 | 6.3 | 37 | 24 | 57 | 17.6 |
|  | Th | 10 | 0.82 | 0.47 | 0.95 | 1.0 | 0.5055 | 0.6 | 1.5 | 9.2 | 2.7 | 1.8 | 4.2 | 25.4 |
| QP | Th | 10 | 0.93 | 0.73 | 0.98 | 0.3 | 0.4396 | 0.2 | 0.4 | 2.8 | 0.8 | 0.5 | 1.2 | 7.8 |
| FHB | Th | 10 | 0.93 | 0.74 | 0.98 | 0.5 | 0.7141 | 0.3 | 0.8 | 3.5 | 1.5 | 0.9 | 2.3 | 9.7 |
| AbDM | Th | 10^a^ | 0.89 | 0.64 | 0.97 | 0.7 | 0.1155 | 0.5 | 1.1 | 6.3 | 2.0 | 1.3 | 3.0 | 17.4 |
| PF_prox_ | Th | 10 | 0.95 | 0.80 | 0.99 | 0.14 | 0.8014 | 0.09 | 0.22 | 4.3 | 0.40 | 0.26 | 0.62 | 12.0 |
| PF_mid_ | Th | 10 | 0.93 | 0.75 | 0.98 | 0.10 | 0.5877 | 0.06 | 0.16 | 4.8 | 0.28 | 0.18 | 0.43 | 13.2 |
| PF_dist_ | Th | 10 | 0.89 | 0.65 | 0.97 | 0.06 | 0.0008 | 0.04 | 0.09 | 5.0 | 0.15 | 0.10 | 0.24 | 13.9 |
| TA_long_ | Th | 10 | 0.68 | 0.17 | 0.91 | 1.3 | 0.5427 | 0.9 | 2.1 | 6.0 | 3.7 | 2.4 | 5.8 | 16.7 |
| TA_trans_ | Th | 10 | 0.63 | 0.09 | 0.89 | 1.3 | 0.8661 | 0.9 | 2.0 | 6.0 | 3.7 | 2.4 | 5.7 | 16.6 |
| FDL | Th | 10 | 0.95 | 0.82 | 0.99 | 13 | 0.3156 | 9 | 21 | 9.3 | 37 | 24 | 58 | 25.9 |
| PER_long_ | Th | 10 | 0.84 | 0.49 | 0.96 | 1.0 | 0.0050 | 0.6 | 1.6 | 7.2 | 2.8 | 1.8 | 4.3 | 19.9 |
| PER_trans_ | Th | 10 | 0.87 | 0.59 | 0.97 | 0.9 | 0.1538 | 0.6 | 1.4 | 6.9 | 2.6 | 1.6 | 4.0 | 19.0 |
| FHL_long_ | Th | 8 | 0.93 | 0.61 | 0.99 | 1.0 | 0.0501 | 0.6 | 1.6 | 3.9 | 2.6 | 1.6 | 4.3 | 10.8 |
| FHL_trans_ | Th | 10 | 0.98 | 0.91 | 0.99 | 0.6 | 0.9434 | 0.4 | 0.9 | 2.7 | 1.7 | 1.1 | 2.6 | 7.3 |

# *ICC: intra-class correlation coefficient, SEM: standard error of measurement, SDC: smallest detectable change, CI- and CI+: lower and upper limit of the 95% confidence interval, CSA: cross-sectional area, th: thickness, AbH: m. abductor hallucis, FDB: m. flexor digitorum brevis, QP: m. quadratus plantae, FHB: m. flexor hallucis brevis, AbDM: m. abductor digiti minimi, PF: plantar fascia, prox: proximal, mid: middle, dist: distal, TA: m. tibialis anterior, long: longitudinal, trans: transversal, FDL: m. flexor digitorum longus, PER: m. musculus peroneus, FHL: m. flexor hallucis longus. The units of measurement of the SEM and SDC are mm (thickness), mm^2^ (CSA), or a percentage of the group mean muscle size (%SEM and %SDC). ^a^ indicates a non-normal distribution of the difference between the repeated measurements. p-value is presented for the comparison across machines.*
