# Supplementary material for: Intra-assessor reliability and measurement error of ultrasound measures for foot muscle morphology in older adults using a tablet-based ultrasound machine
Source: J Foot Ankle Res. 2022 Jan 25;15:6. doi: 10.1186/s13047-022-00510-1 (PMC8788121; doi:10.1186/s13047-022-00510-1)
Supplement: Supplementary file 4 — Additional file 4. Graphical presentation of the raw data on which the measurement properties are based. [file 13047_2022_510_MOESM4_ESM.docx]

#
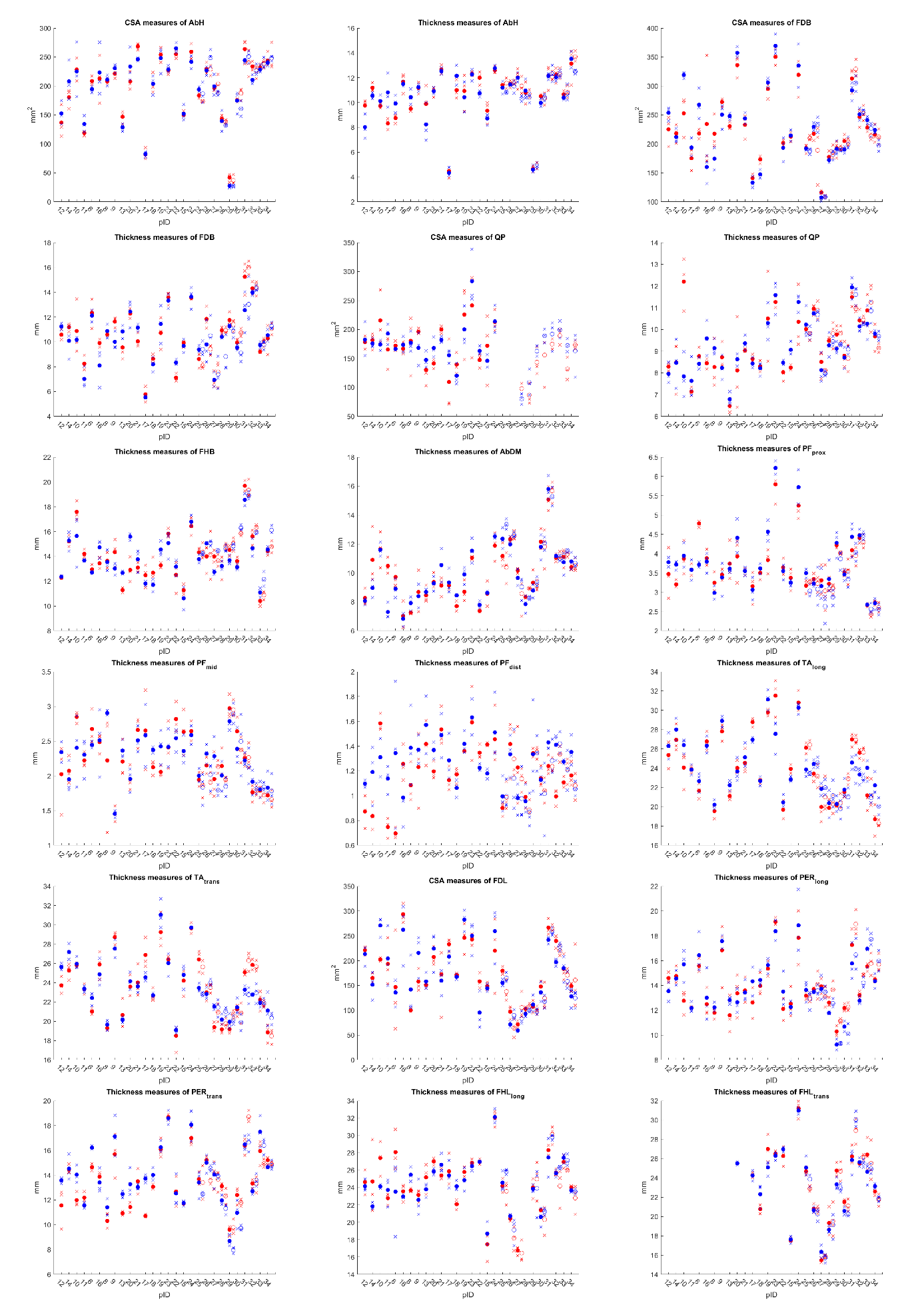


***Raw data.*** *CSA: cross-sectional area; AbH: m. abductor hallucis, FDB: m. flexor digitorum brevis, QP: m. quadratus plantae, FHB: m. flexor hallucis brevis, AbDM: m. abductor digiti minimi, PF: plantar fascia, prox: proximal, mid: middle, dist: distal, TA: m. tibialis anterior, long: longitudinal, trans: transversal, FDL: m. flexor digitorum longus, PER: m. musculus peroneus, FHL: m. flexor hallucis longus, red: occasion 1, blue: occasion 2; cross markers: trials, circle markers: average morphology measure per participant, filled markers: tablet-based machine, non-filled markers: mainframe machine.*
